# From ductal carcinoma in situ to invasive breast cancer: the prognostic value of the extracellular microenvironment

**DOI:** 10.1186/s13046-024-03236-z

**Published:** 2024-12-23

**Authors:** Taylor S. Hulahan, Peggi M. Angel

**Affiliations:** Department of Pharmacology and Immunology, Medical University of South Carolina, Charleston, SC USA

**Keywords:** Extracellular matrix (ECM), Tumor microenvironment, Ductal carcinoma in situ (DCIS), Invasive breast cancer (IBC), Collagen, Glycosylation, Proteomics, Biomarker

## Abstract

Ductal carcinoma in situ (DCIS) is a noninvasive breast disease that variably progresses to invasive breast cancer (IBC). Given the unpredictability of this progression, most DCIS patients are aggressively managed similar to IBC patients. Undoubtedly, this treatment paradigm places many DCIS patients at risk of overtreatment and its significant consequences. Historically, prognostic modeling has included the assessment of clinicopathological features and genomic markers. Although these provide valuable insights into tumor biology, they remain insufficient to predict which DCIS patients will progress to IBC. Contemporary work has begun to focus on the microenvironment surrounding the ductal cells for molecular patterns that might predict progression. In this review, extracellular microenvironment alterations occurring with the malignant transformation from DCIS to IBC are detailed. Not only do changes in collagen abundance, organization, and localization mediate the transition to IBC, but also the discrete post-translational regulation of collagen fibers is understood to promote invasion. Other extracellular matrix proteins, such as matrix metalloproteases, decorin, and tenascin C, have been characterized for their role in invasive transformation and further demonstrate the prognostic value of the extracellular matrix. Importantly, these extracellular matrix proteins influence immune cells and fibroblasts toward pro-tumorigenic phenotypes. Thus, the progressive changes in the extracellular microenvironment play a key role in invasion and provide promise for prognostic development.

## Background

### Epidemiology of DCIS

Ductal carcinoma in situ (DCIS) represents 20% of breast cancer diagnoses in the United States with a rising incidence attributed to increased mammographic screening [1]. From misdiagnosis studies, 14–53% of untreated DCIS patients are estimated to develop invasive breast cancer within 10–15 years following their primary DCIS diagnosis [2]. Despite this progression risk, the current therapeutic paradigm remains one of overtreatment. DCIS patients can undergo surgical removal of the tumor and surrounding breast tissue, endocrine therapy dependent on hormone receptor status, and radiation therapy. These therapeutic interventions are not without risks including coronary events, pulmonary dysfunction, axillary discomfort, and, although rare, the development of secondary therapeutically induced cancers [1]. Notably, a Sloan-Kettering Cancer Center study sampling 271 patients with tumor sizes less than 2 mm and a fraction with positive surgical margins found that 60% of patients who did not undergo radiation therapy or endocrine ablative therapy were alive and had no evidence of IBC 20 years following their initial DCIS diagnosis [3]. In consideration of patient preference, the LORD trial offered low-risk DCIS patients, defined by clinicopathological features, the choice between active surveillance and conventional therapy. Interestingly, 76% of patients chose active surveillance [4]. These findings suggest that risk stratification has the potential to change the current treatment paradigm. However, prognosticators are needed that can better stratify patients into high and low-recurrence risk categories.

Specific patient demographics have been studied in DCIS patients with the intention to better identify patient populations at increased recurrence risk. Such clinical characteristics include menopausal status and body mass index. Given the association between younger age at diagnosis and increased breast cancer aggressiveness [5, 6], it is perhaps unsurprising that the Shamliyan et al. 2010 and Visser et al. 2019 studies reported that premenopausal women with DCIS had increased rates of ipsilateral IBC recurrence than their postmenopausal counterparts [7, 8]. Although the body mass index is a well-known risk factor of breast cancer, there have been limited studies on body mass index and recurrence in DCIS patients. Shamliyan et al. 2010 documented an increased ipsilateral IBC recurrence risk for DCIS patients in the overweight and obese categories.

Race and ethnicity have been associated with a varied risk of IBC progression. Black women with DCIS are more likely to recur and develop invasive breast cancer subsequently [7–10]. A study of 163,892 women (10.5% black, 9.8% Asian, and 8.6% Hispanic) found significantly increased rates of IBC recurrence in black women with a hazard ratio of 1.42 (1.32–1.52) compared to their white counterparts. Notably, black women within this study were found to be at a significantly increased recurrence risk by gene expression compared to white patients [9]. Importantly, the racial disparity in recurrence has not been explained by differences in therapeutic intervention [9, 10]. While these studies highlight racial disparities in DCIS progression, there have been limited studies on molecular patterns that could vary between these patient populations and contribute to the disparities in clinical outcomes.

### Clinicopathology of DCIS

Clinicopathological features remain the mainstay for recurrence risk assessment. High nuclear grade has been found to be predictive of recurrence including that of invasive breast cancer [7, 8, 11, 12]. In long-term follow-studies, 35% of DCIS patients with low-grade disease progress to IBC over 50 years, and 50% of DCIS patients with high-grade lesions progress to IBC over 3 years [1]. Comedo necrosis, defined by the presence of a central necrotic region within the ductal lumen, has been reported to be significantly associated with general recurrence [7, 12], and associated, albeit not significantly, with invasive breast cancer recurrence [8, 11]. Although these histological features have notable predictive value, consistent histological evaluation as risk assessment remains a clinical challenge [13, 14].

Molecular profiling for hormone receptor status and HER2 amplification is routinely performed for DCIS. Approximately 70% of DCIS cases are classified as estrogen receptor-positive and progesterone receptor-positive [15–17] while HER2 expression is reported in approximately 30% of cases [18]. Estrogen receptor positivity has been linked to lower recurrence incidences reaching significance in certain studies [7, 11, 12, 19]. Although not found to be significant, progesterone receptor positivity has been associated with increased recurrence including that of invasive breast cancer. Interestingly, HER2 amplification has been found to be significantly correlated with overall recurrence [7, 12] but was not significant for invasive breast cancer recurrence [8, 11]. DCIS patients with palpable tumors that were COX-2, Ki67, and p16 positive had significantly higher eight-year IBC recurrence risk [20]. Notably, high-grade DCIS lesions had significantly higher COX2 staining in the normal adjacent epithelium than low-grade lesions. Beyond immunohistochemical findings, additional features such as positive surgical margins [7, 8, 11, 12] and tumor size had demonstrated positive correlations with recurrence [7, 12]. A retrospective study of 71 DCIS patients demonstrated that MRI features including those of the lesion and background parenchymal enhancement following six months of endocrine therapy could stratify patients into low, intermediate, and high IBC recurrence risk. This study found that 8.7% of low-risk patients and 68.2% of high-risk patients experienced a later IBC event [21].

Composite metrics of clinicopathological features such as the Van Nuys Prognostic Index, DCISionRT, and the Memorial Sloan Kettering nomogram have demonstrated predictive potential. The Van Nuys Prognostic Index (consisting of nuclear grade, tumor size, margin width, necrosis, and patient age) reported increased recurrence rates in DCIS patients classified as high-risk [7, 22]. However, the clinical utility of this index has been debated. Kunkiel et al. 2021 study found that the values for clinical parameters used to assign the index score did not align well with contemporary research regarding tumor size, margin width, and age. Additionally, the low recurrence risk group when treated in compliance with the Van Nuys Index recommendation of local excision alone had a 28.8% recurrence rate over 15 years. Alternatively, DCISionRT combines immunohistochemical staining and scoring of COX2, FOXA1, HER2, Ki-67, p16/CDKN2A, PR, and SIAH2 in addition to clinical characteristics (age, tumor size, margin status, and palpability) [23]. In the Bremer et al. 2018 study on DCISionRT, the low decision score group had a 4% 10-year IBC recurrence risk while the elevated decision score group had a 15% risk. While this study demonstrated the predictive value of DCISionRT in identifying an elevated risk group that could benefit from the addition of radiotherapy, this elevated risk group had only a 23% 10-year risk of IBC when treated with breast conserving surgery alone [24]. Unlike the previous composite metrics, the Memorial Sloan Kettering Cancer Center (MSKCC) DCIS Nomogram evaluates a DCIS patient’s recurrence risk using age at diagnosis, family history, clinical or radiological presentation, use of adjuvant radiation or endocrine therapy, nuclear grade, presence of necrosis, positive or close surgical margins, the number of surgical excisions, and the year of surgery. In Yi et al. 2012 study of 734 DCIS patients, the MSKCC nomogram was reported to have a predictive accuracy of 0.634 evaluated by area under the receiver-operator curve analysis and to overestimate the risk of recurrence in the high-risk group [25].

Overall, clinicopathological characteristics provide the most utilized and currently predictive features for recurrence risk stratification. Evaluation of multiple features in composite metrics seems to increase the predictive potential of the clinicopathology in DCIS. However, consistent pathological evaluation of DCIS remains a challenge [13, 14], and only a small proportion of DCIS patients in the high-recurrence risk categories seem to recur. Artificial intelligence has improved clinicopathological risk stratification in breast cancer within research settings. The Howard et al. study demonstrated that utilization of deep learning algorithms for histological evaluation and integration with clinical parameters could predict recurrence-free survival and recurrence-free interval comparably to recurrence risk assessed by genomic assays [26]. Furthermore, the use of machine learning techniques to integrate clinical, transcriptomic, genomic, pathological features, and therapeutic intervention of breast cancer patients demonstrated increased accuracy for predicting pathological complete response than the evaluation of a subset of these features [27]. While machine learning has increased the prognostic accuracy of clinicopathological models, applying these approaches to DCIS to improve risk stratification is needed, particularly for therapeutic de-escalation in low-risk patients.

### Genomic assays for recurrence assessment

Genomics studies in breast cancer have been extensive and have led to clinically utilized genomic prognostic assays including MammaPrint^®^, Oncotype Dx^®^, Prosigna^®^, and EndoPredict^®^ [28–38]. Contemporary work with artificial intelligence (AI) and machine learning platforms for the identification of predictive genomic markers has shown great potential. In the Yagin et al. study, a machine learning approach was used to identify an 18-gene signature from 24,481 genes that could predict breast cancer metastasis with an accuracy of 96% [39]. Specific to triple-negative breast cancer, Chen et al. used machine learning to identify 3 genes from 147 differentially expressed between the breast cancer and normal tissue. These 3 genes, BUB1, CCNA2, and PACC1, were linked to decreased disease-free survival [40]. While AI-identified genomic signatures have shown prognostic promise, most have not been focused on DCIS recurrence [39, 40].

Of these aforementioned clinically utilized genomic assays, Oncotype Dx^®^ has been the only one to be adapted to DCIS recurrence. Oncotype Dx^®^ is a gene assay sampling 21 genes that have been demonstrated to predict patient response to chemotherapy and recurrence risk in early-stage invasive breast cancer. Incredibly, 99% of invasive breast cancer patients with low recurrence scores treated with endocrine therapy alone had no evidence of cancer 5 years later [29]. In an effort to develop a DCIS-specific assay, Oncotype Dx^®^ was reduced to 12 genes (Ki-67, STK15, survivin, CCNB1, MYBL2, PR, GSTM1, ACTB, GAPDH, RPLPO, GUS, and TFRC) to create the DCIS recurrence score. In subsequent studies, 25.9% and 19.2% of DCIS patients with high recurrence scores eventually recurred or were found to have a subsequent invasive breast cancer event respectively. Notably, 10.6% and 3.4% of DCIS patients with low recurrence risk scores were found to recur or progress to IBC respectively [41]. Although the DCIS recurrence score reports increased recurrence rates within the high recurrence score groups, a large portion of the DCIS patients in this subset did not recur. Overall, this might suggest additional genes could be added to the genomic assay or clinicopathological metrics may further improve recurrence risk stratification.

### Stromal markers as predictors of DCIS progression

Contemporary work in the DCIS field has begun to focus on the microenvironment for predictors of disease progression. Risom et al. 2022 study discovered discontinuity of the myoepithelial layer, increased immune infiltrate, and decreased collagen density in patients who did not progress to IBC (non-progressors) compared to ipsilateral progressors (DCIS patients who experienced IBC recurrence). Here, a comparison of the primary DCIS event and the IBC recurrence from the sample patients revealed decreased normal fibroblast populations with IBC recurrence. Further, IBC recurrences were found to have increased cancer-associated fibroblast populations and increased collagen density [42]. In subsequent work, Strand et al. 2023 demonstrated four distinct clusters based on transcriptional expression of stromal genes (desmoplastic, collagen-rich, normal-like, and immune-dense). Of these four clusters, the DCIS patients classified as the desmoplastic group had a significantly longer time to progression than patients classified as other clusters [43].

While historically clinicopathological evaluation and genomic screening assays have been utilized for recurrence risk stratification, spatial research has increasingly begun to demonstrate the important role of the extracellular microenvironment in the progression to IBC. These studies highlight the potential of stromal markers in predicting IBC progression and underscore the continued investigation of stromal signatures in DCIS.

## Cellular composition of DCIS stroma

**Fig. 1:**
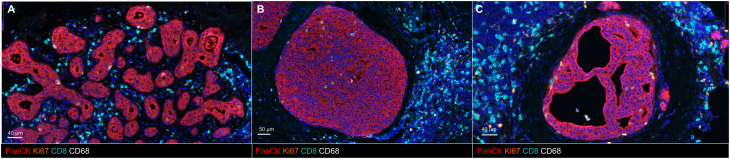
Lymphocyte-rich ECM Microenvironment surrounding DCIS lesions. (**A**) Multiplex immunohistochemistry identifies immune infiltrate as predominantly killer T-cells within the local ECM microenvironment. (**B**) Multiplex immunohistochemistry demonstrates adjacent lymphocytic infiltrate surrounding an example solid DCIS lesion. (**C**) A lymphocytic population is reported to surround an example cribriform lesion. In all images, blue marks the nuclear counterstain.

The normal breast microenvironment consists of ductal cells separated by a myoepithelial layer from the immune cells and fibroblasts residing within a collagen-rich extracellular matrix [44]. With malignant transformation, the myoepithelial barrier breaks down and interaction between these cell types and the extracellular matrix leads to further dysregulation and disease progression [45]. Here, fibroblasts and immune cells, key cellular components of the stroma, are highlighted for their role in the production and remodeling of the extracellular microenvironment in addition to their prognostic value in DCIS (Fig. 1).

### Fibroblasts secrete and remodel the ECM in DCIS

Cancer-associated fibroblasts (CAFs) play a key role in extracellular matrix (ECM) remodeling [46, 47] and are crucial in DCIS’ progression to IBC [48]. Importantly, CAFs contribute to the formation of collagen “highways” for invasive cancer cells to travel out of the primary tumor site [49–51]. Specific CAF subsets have been linked to invasion [52, 53] suggesting their specific role in the invasive transformation of DCIS. Interestingly, DCIS fibroblasts have been found to be transcriptionally distinct from invasive ductal carcinoma (IDC) and normal fibroblasts demonstrating upregulation of invasion-related genes [54]. In addition to their capability to remodel collagen organization to support invasion, CAFs are a dominant source of aberrant collagen expression. Giussani et al. demonstrated that CAFs produced elevated levels of breast cancer-associated collagen types XI and Xα1 [55]. Similarly, Risom et al. reported high CAF density to be associated with increased collagen and other extracellular matrix protein expression. In an effort to connect CAF density to patient outcomes, an investigation of recurrence events in the same study revealed that normal fibroblast density decreased within the IBC recurrence event [42]. Another recurrence study of primary DCIS events found myofibroblasts (PDGFRa+, αSMA + cells) were significantly higher in DCIS that recurred than those that did not [56]. Given its association with invasion and recurrence, CAFs have been clinically explored as biomarkers in DCIS. FAP-a and PDGFRβ have been positively correlated with increased IBC recurrence [57, 58]. However, their use as biomarkers has challenges [59, 60] due to the heterogeneity of CAF populations in primary tumors [52, 61–63]. Larger, multi-institutional studies are required to validate these discrete CAF markers for their prognostic value in DCIS before clinical integration is achieved. Investigation of these heterogenous CAF populations in relation to the surrounding collagen matrix will provide additional insights into DCIS microenvironments that are at high risk for invasive recurrence.

### Tumor-associated macrophages are reprogrammed by the extracellular matrix

The extracellular matrix and tumor-associated macrophages (TAMs) interact within the microenvironment to influence tumor biology. While macrophages remodel the extracellular matrix, the extracellular matrix also modifies their function and infiltration [64]. Macrophages can mechano-sense the collagen matrix through their integrin receptors, which helps direct their migration throughout the matrix [65]. In DCIS, macrophages become more abundant than in normal breast tissue [66]. In addition to quantifying macrophage infiltration, defining the macrophage types has become increasingly important. Macrophages are canonically categorized as M1, anti-tumorigenic, or M2, pro-tumorigenic [67]. In in vivo studies, collagen of non-specific type has been shown to stimulate M2 polarization [68]. Particularly, increased collagen density has been associated with different transcriptional profiles of cytokines and immune regulatory genes of TAMs. Within this study, transcripts involved in the recruitment of CD8 + T cells were reduced while those involved in regulatory T cell recruitment were increased [69]. This highlights the significance of collagen-macrophage interactions in orchestrating an immunosuppressive microenvironment that favors breast cancer progression. In a comparative study of DCIS and IDC, Acerbi et al. reported increased M2 macrophages at the invasive front of IDC. Here, increased macrophage infiltration was positively correlated with a stiffer extracellular matrix [70]. Taken together, this could implicate macrophages as key remodelers of the extracellular matrix at the invasive border in IDC.

Clinically, increased TAM infiltration is associated with poor patient prognosis in breast cancer except in relatively small estrogen receptor-positive tumors [71–74]. CD68-positive macrophages are significantly elevated in high-grade DCIS compared to non-high-grade DCIS [75]. Considering the defined pro-tumorigenic effects of M2 macrophages, CD163, an M2 marker, has been explored in DCIS recurrence. The high density of CD163-positive macrophages has been correlated with increased ipsilateral invasive breast cancer recurrence and high-risk histological features such as high nuclear grade and microinvasion [76]. Furthermore, CD163-to-CD68 ratios above 0.46 were associated with a significantly decreased probability of disease-free survival in DCIS patients [77]. Thus, TAMs, specifically of the M2 phenotype, have been linked to invasion and demonstrated clinical utility in the prediction of DCIS recurrence. While translational studies in DCIS have primarily focused on CD163 as an M2 marker, TAMs are phenotypically diverse and may be further divided into subtypes with different functions [78]. Understanding these subtypes requires multiplex imaging that is not utilized in the current clinical DCIS work-up. It is important to note that the entire tumor microenvironment including the spectrum of polarized TAMs can drive breast cancer progression. Studying the adjacent collagen composition might further elucidate why M2 reprogramming occurs and identify alternative targets for recurrence prevention.

### Tumor-infiltrating lymphocytes promote invasion and modify the collagen matrix

Tumor-infiltrating lymphocytes (TILs) can consist of T regulatory cells, killer T cells, helper T cells, and B cells. Together, they can contribute to an immunosuppressive microenvironment that can facilitate invasion [79]. An in vivo study of the murine mammary tumor virus polyoma middle T (MMTV-PyMT) model demonstrated that T regulatory cell ablation at the in situ stage led to increased tumor burden and progression to IBC. Here, T regulatory cell ablation was linked to increased collagen deposition and desmoplasia [79]. TILs were similarly linked to collagen alterations in the Toss et al. study, in which increased COL11A1 expression was associated with dense TIL infiltrate [80]. Taken together, T-cells, particularly regulatory T cells, modulate the surrounding collagen matrix and contribute to invasion.

Clinically, TILs have been investigated for their predictive value in DCIS. Farolfi et al. found that increased TILs associated with high-risk histological features including necrosis and high nuclear grade. While increased TIL number also was correlated to increased ipsilateral secondary breast cancer events, this was not found significant [81]. Pruneri et al. similarly found no significant association between TILs and secondary breast cancer events [82]. This lack of association could be due to the importance of TIL localization for invasion. In a subsequent study, increased density of touching TILs, those abutting or within one lymphocyte’s width from the basement membrane, was associated with a significantly higher five-year rate of ipsilateral invasive breast cancer recurrence than patients with less dense touching TILs [83]. A high number of touching TILs (≥ 10 per duct) has also been linked to upstaging low-grade DCIS to high-grade DCIS or IBC [84].

The composition of the TIL population also likely influences the pro-invasive DCIS microenvironment. CD4+, CD8+, and FoxP3 + T cells can be present throughout the stroma. Studies have shown that increased infiltration of FoxP3+, CD4+, and CD8 + cells associated with high-grade DCIS [75, 85]. Furthermore, a high CD4+-to-CD8 + ratio associated with an increased risk for ipsilateral invasive recurrence [86]. In addition to increased CD8 staining, synchronous DCIS-IBC cases were shown to have increased stromal staining of PD-1 and CD20 compared to pure DCIS cases. High stromal PD-1 expression was linked to shorter local recurrence-free intervals including invasive recurrences. Interestingly, within this same study, TIL density was reported to be the greatest predictor of IBC recurrence in a multivariate analysis [87]. In a B cell-focused study, high para- and peri-tumoral B lymphocytes were associated with shorter recurrence-free survival [88]. Thus, specific lymphocytic infiltration has shown prognostic value in DCIS recurrence.

In addition to lymphocytic composition, complex lymphocytic formations have been investigated for their prognostic value in DCIS. Tertiary lymphoid structures (TLS) are defined by the inner region of CD20-positive B cells surrounded by CD3-positive T cells, which can consist of helper, cytotoxic, or regulatory T cells [89]. Although the presence of TLSs has often been associated with improved patient outcomes [89], increased TLSs in DCIS have been linked to higher-risk clinical features such as larger tumor size, necrosis, and high nuclear grade. However, increased TLSs have not been associated with invasive recurrence [90]. In fact, Acar et al. found lower numbers of TLSs to be associated with better overall survival [91]. These aforementioned studies underscore the need for additional studies into the spatial distribution and composition of TLSs in relation to breast cancer invasion. While TILs have been extensively studied for their prognostic value in DCIS, the spatial distribution, organization, and composition of TILs should be further investigated to understand their influence on DCIS tumor biology and patient outcomes. This requires a multiplex imaging approach and spatial computational analysis that is not yet amenable to the clinical workflow.

**Fig. 2:**
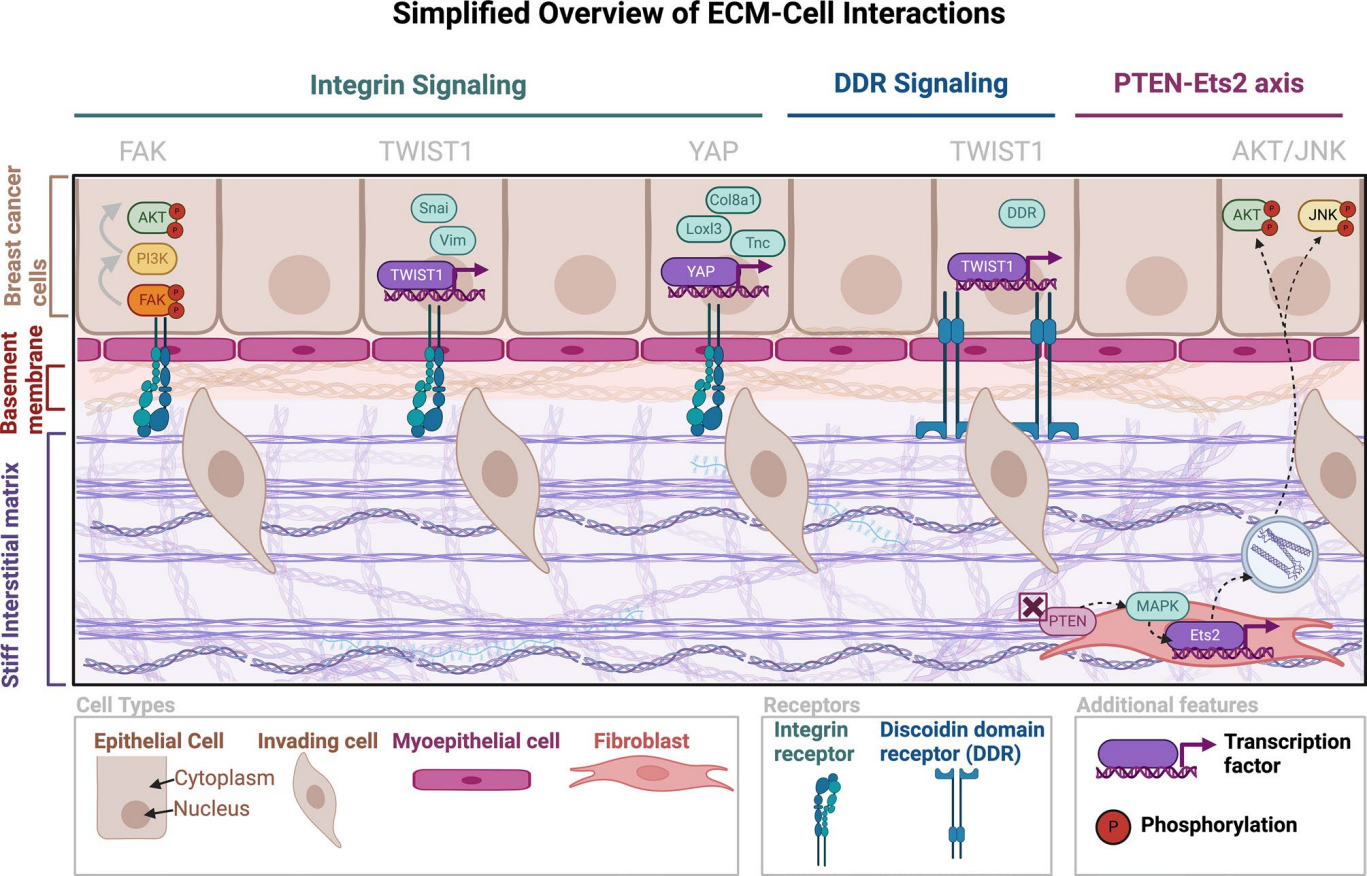
The Extracellular Matrix Stiffness Promotes Tumor Cell Invasion. (**A**) A simplified schematic of how stiff matrix can elicit invasive phenotype through β1 integrin and discoidin domain receptor-dependent manners

### Collagen matrix stiffness drives an invasive phenotype in tumor cells

Extracellular matrix stiffness is crucial in facilitating invasion through the promotion of the epithelial-to-mesenchymal transition (EMT; Fig. 2). Breast epithelial cells mechanosense their microenvironment through collagen-binding receptors such as discoid domain receptors (DDR) and integrin receptors [92, 93]. Binding through integrin and discoidin domain receptors can elicit nuclear translocation of discrete transcription factors and activate EMT transcriptional programs [94–96]. In a β_1_ integrin-dependent manner, a stiff collagen matrix can lead to TWIST1 nuclear translocation [97]. Similarly, increasing collagen cross-linking through the upregulation of lysyl oxidase has demonstrated focal adhesion kinase activation through β_1_ integrin and led to phosphoinositide 3-kinase (PI3K)-AKT pathway activation. This promoted an invasive phenotype in an erythroblastic oncogene B (ErbB2)-positive model [98]. Also modulated through β_1_ integrin signaling, YAP nuclear localization is thought to lead to EMT and invasion [99]. In the context of collective invasion, YAP nuclear localization is reportedly increased in leader cells in basal breast cancer organoid models cultured in a collagen type I matrix and led to the upregulation of ECM-related genes including Tnc, Col8a1, and Loxl3 [100]. Given that collagen type I is not a constituent of the basement membrane, a 3D model using reconstituted basement membrane and alginate instead of a collagen type I matrix has been used to model DCIS. In this 3D model, increased stiffness did not result in YAP nuclear localization [101]. These differences are likely due to compositional differences in the matrix of the culture system used. To avoid artifacts from model systems, additional studies are necessary to report the spatial localization of discrete collagen types and their association with known transcription factors in mechanotransduction in human specimens.

Similar to their β_1_ integrin counterpart, DDRs have been implicated in breast cancer progression. DDR1 is normally expressed in mammary epithelial cells and has been found to be crucial in cell migration [93]. Notably, DDR1 knockout in the PyMT murine model led to increased DDR2 expression presumed to be through expansion of the myoepithelial cell compartment. Takai et al. demonstrate loss of DDR1 leads to an aggressive basal phenotype with increased metastasis. While most DCIS cases are of the luminal subtypes, the study underscores the importance of DDR1-ECM interactions in controlling breast cancer progression [102]. In the context of breast cancer recurrence, DDR2 expression has been demonstrated to be elevated in recurrent breast cancers compared to non-recurrent cases. Overexpression of TWIST1 elicited increased DDR2 expression, which suggests that EMT transcriptional programs upregulate DDR2 expression. In the presence of collagen, DDR2 was found to increase tumor cell viability, emphasizing the importance of tumor responsiveness to the adjacent matrix in breast cancer progression [103]. While further work is needed to characterize the surrounding composition of the collagen matrix and its effect on integrin and DDR signaling, it is well understood that the collagen matrix can polarize the breast epithelium toward an invasive phenotype.

Stromal PTEN loss has also been linked to breast cancer progression, particularly invasion [104, 105]. In stromal fibroblasts, PTEN ablation led to Est2 nuclear translocation activating both AKT and JNK pathways [105]. Adjacent ductal epithelium was also found to have these pathways activated which was attributed to the surrounding ECM remodeling and increased ECM deposition from mutated fibroblasts [104, 105]. These ECM changes were further associated with an invasive phenotype in ductal cells [104]. Thus, breast epithelial cells are responsive to changes in their environment adapting to become more invasive with increasing ECM deposition and altered organization. To better understand the transition from non-invasive to invasive breast pathologies, further studies are necessary to fill in the gaps in the signaling cascades elicited by ECM alterations.

## Collagen regulation in DCIS

### Links between breast pathologies and collagen structural organization

With malignant transformation, the ECM stiffens, which is thought to be primarily mediated through alterations to collagen. The transformation of normal breast epithelium to premalignant tumors has been characterized by an increase in collagen abundance using visualization and quantification by picrosirius red staining. Throughout this malignant transformation, there is a progressive increase in tumor elastic modulus [98, 106]. This progressive stiffening has been associated with increased lysyl oxidase-mediated collagen crosslinking within the tumor. Inhibition of lysyl oxidase has been shown to delay tumor progression, decrease tumor burden, and preferentially lead to low-grade tumor formation using the MMTV-Neu murine models for breast cancer progression [98].

It is important to note that collagen fiber organization also contributes to the mechanical properties of the matrix [107]. Collagen fiber alignment has been well characterized in breast cancer as tumor-associated collagen signatures (TACS). As breast cancer progresses, collagen fiber density increases (TACS-1), aligns parallel to the tumor border (TACS-2) and subsequently realigns perpendicular to the tumor border (TACS-3) [108–110]. Notably, TACS-3 has been associated with invading tumor cells, and tumor cells are known to remodel their surrounding collagen matrices to promote this invasion [109]. In in vivo studies, high collagen density has been associated with progression to TACS-3 over 10 weeks, increased invasion, and lung metastases [108]. In a 207-patient cohort, TACS-3 was found to be associated with decreased disease-free and disease-specific survivals [110]. A DCIS-specific study following 227 women reported that perpendicular collagen fiber alignment was more prevalent in patients who had comedo necrosis and those who were estrogen receptor-negative, progesterone receptor-negative, and HER2-positive. However, in this study, collagen fiber alignment was not found to be associated with recurrence [111]. Although no association was found between alignment and recurrence in DCIS, Sprague et al. showed that greater fiber width was linked to decreased recurrence while straightened fibers and increased distance between two fibers were positively associated with recurrence [112]. Interestingly, Xi et al. found that TACS and TACS corresponding microscopic features could discriminate between histological grade 1 and 2 or 3 using a training cohort of 328 breast cancer patients and a validation cohort of 215 breast cancer patients. TACS independently could distinguish between histological grades with an area under the curve above 0.72 in the training and validation cohorts [113].

While alterations in collagen organization and mechanical properties of the matrix are understood to be important in disease progression, this has yet to be clinically applied in breast cancer. Collagen-specific probes for magnetic resonance imaging (MRI) have demonstrated diagnostic potential in prostate cancer through the characterization of collagen changes associated with cancer and accurate tumor localization [114]. Currently, a breast tumor-specific collagen architecture is acknowledged to be linked to survival outcomes and invasion, but this knowledge has remained clinically underutilized.

**Fig. 3:**
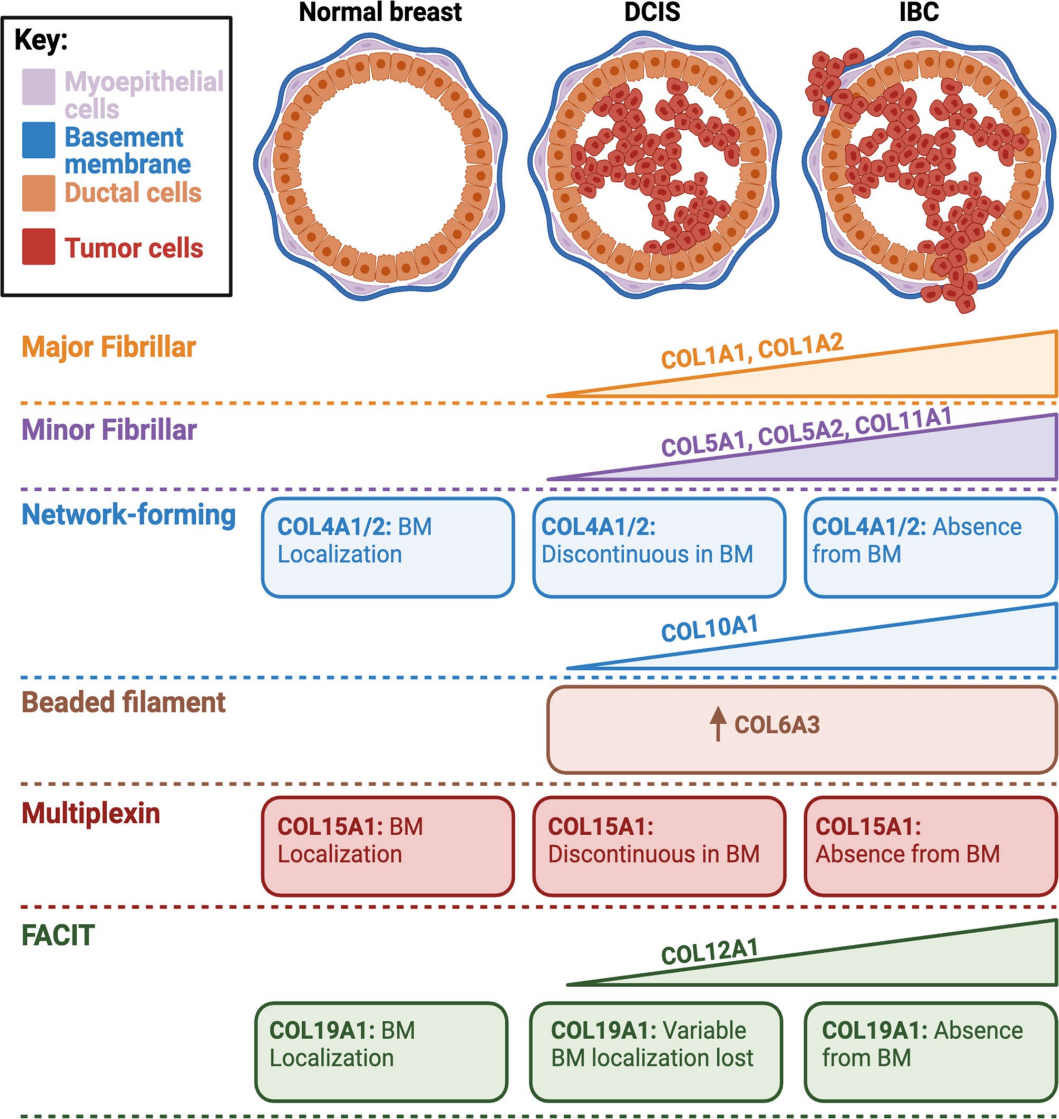
Collagen Alterations Linked to Malignant Transformation. Many collagens demonstrate increasing expression and changes in localization with the progression to IBC

### Breast cancer and collagen stroma variation

Collagen is the most abundant component of the interstitial matrix in the normal breast microenvironment. In breast cancer, many collagens increase in abundance and contribute to discrete stages in breast cancer progression (Fig. 3; Table 1) [108]. Numerous studies have focused on the class of fibrillar collagens defined by their uninterrupted triple helical domains [115–126]. Major fibrillar collagens such as type I and III have been shown to be crucial in invasion and metastasis. Importantly, in vitro studies have shown that COL1A1 knockdown reduced invasion and metastases [127]. The clinical relevance of COL1A1 has been underscored by contemporary translational studies. High cytoplasmic protein expression of the ⍺1 chain of collagen type I has been associated with worse overall survival of estrogen receptor-positive breast cancer patients [127]. In a The Cancer Genome Atlas (TCGA) comparative study between normal breast tissue (*n* = 113) and breast tumors (*n* = 1109), COL11A1, COL12A1, COL1A1, COL1A2, COL5A1, and COL5A2 transcripts were upregulated in breast cancer and could discriminate between the two breast tissues. Notably, COL1A1 and COL11A1 transcripts demonstrated high discrimination between normal breast tissue and breast tumors [124]. Another major fibrillar collagen, collagen type III, has been shown to be transcriptionally upregulated in dormant murine breast cancer models and tumor-restrictive or pro-invasive depending on cell-specific expression [128–130]. A fibroblast-specific knockdown of COL3A1 led to collagen alignment patterns linked to poor prognosis and elicited an aggressive breast epithelial cell morphology including in TNBC cell lines. Interestingly, higher COL3A1:COL1A1 expression was found in non-invasive regions compared to invasive breast pathologies and associated with better survival outcomes in the TCGA cohort [129]. Although it is uncommon for DCIS to be classified as TNBC, it is important to note that COL3A1 expression in cancer cell lines has been demonstrated to be promote invasion in TNBC, which perhaps underscores the role of cell-specific expression in modulating tumor phenotype [130]. These findings coincide well with a proteomic comparison using liquid chromatography-tandem mass spectrometry between matched breast disease (fibroadenoma, DCIS, and IBC) and normal breast tissue from ten patients, which revealed significant increases in the fibrillar collagen class in DCIS and IBC including in collagen type III [131]. While these studies support major fibrillar collagens’ role in mediating malignant transformation, further investigations are necessary to understand the seemingly divergent roles of collagen type III in breast progression and why expression in certain cell types might induce different phenotypes. Larger human studies are needed to evaluate the prognostic value of COL1A1 and COL3A1 in DCIS recurrence to be clinically utilized as biomarkers.

In addition to the major fibrillar collagen class, many minor fibrillar collagens, including those aforementioned (COL11A1, COL5A1, and COL5A2) have reported prognostic value in breast cancer. Although less abundant than their major fibrillar counterparts, minor fibrillar collagens contribute to collagen fibril formation and have been generally found to increase from benign to invasive breast disease [132]. Specifically, elevated protein expression of the α1 chain of collagen type V has been demonstrated in IDC (*n* = 90) compared to the benign breast pathology, fibroadenoma (*n* = 90). High COL5A1 expression positively correlated with estrogen receptor and progesterone receptor expression [122]. In TNBC, high COL5A1 expression has been associated with chemoresistance and poor survival outcomes. Silencing COL5A1 in a chemo-resistant MDA-MB231 cell line led to re-sensitization to doxorubicin. Interestingly, COL5A1 immunohistochemical staining correlated with CD163, the M2 macrophage marker, which could suggest its role in M2 polarization [121]. Collagen type XI has demonstrated its predictive potential throughout many breast cancer studies [80, 123, 125, 126, 133]. In a 72-patient investigation of the α1 chain of collagen type XI, breast pathologies had decreased stromal staining compared to normal adjacent tissue, specifically for splice forms V1a and Npp. Decreased cytoplasmic staining of the V1a splice form of the α1 chain of collagen type XI in the cancer cells was linked to lymph node metastases [133]. However, subsequent larger studies have reported increased immunohistochemical staining in breast cancer compared to normal breast epithelium [80, 123, 134]. Notably, these investigations did not probe for specific splice forms and evaluated normal breast epithelium and not phenotypically normal adjacent tissue to the cancerous field. A comparative study between invasive carcinomas and non-invasive breast tumors demonstrated that 94% of invasive breast tumors (*n* = 105) demonstrated positive staining for the pro-α1 chain of collagen type XI in surrounding fibroblasts compared to only 3 in situ lesions that stained positive. Notably, collagen type XI staining was better able to identify microinvasion than the myoepithelial markers, calponin, and p63 [126]. In the Toss et al. study [80], increased immunohistochemical staining was discovered in mixed DCIS-IBC (*n* = 239) cases compared to the pure DCIS cases (*n* = 776) with invasive regions reporting the highest expression of COL11A1. High stromal COL11A1 expression was associated with decreased recurrence-free survival specifically invasive breast cancer recurrence in the pure DCIS subset [80]. Taken together with increased M2 macrophages seen with high COL11A1 expression, this could suggest the role of COL11A1 in creating a pro-invasive environment in which immune cells are polarized towards a pro-tumorigenic phenotype and can no longer control the invasion of abnormal ductal cells. In estrogen receptor-positive breast cancers, COL11A1 has been shown to be important in maintaining estrogen receptor signaling and proliferation in tamoxifen-resistant cell lines [135]. As most DCIS cases are estrogen receptor-positive with tamoxifen being a therapeutic option, COL11A1-estrogen receptor interactions need to be investigated as a potential mechanism of therapeutic resistance and recurrence. In the minor fibrillar collagen class, collagen type XI has demonstrated great promise as a prognosticator for DCIS recurrence; however, it has yet to be clinically integrated. A large prospective, multi-institutional study is necessary to validate the association between recurrence and collagen type XI in DCIS as well as to establish if it can benefit current therapeutic decision-making.

The nonfibrillar class of network-forming collagens has also been implicated in breast cancer progression. Network-forming collagens include types IV, VIII, and X and are known for their linear aggregations forming networks [136]. In breast cancer, collagen type IV and X have been primarily investigated for their role in invasive disease [137–140]. Particularly, collagen type IV is of interest in invasive transformation as a constituent of the basement membrane which breaks down in invasion. In an investigation of 22 breast tumors, a subset of the DCIS lesions was found to have discontinuous basement membranes reported by patchy laminin and type IV collagen staining [141]. Considering that discontinuity of the myoepithelial barrier in DCIS has been associated with a decreased risk of progression to IBC [42], this could suggest collagen type IV as a crucial mediator of invasion. In the IBC SKBR3 cell line, collagen type IV was identified as a top differentially expressed gene with COL4A1 knockdown demonstrating its important role in proliferation. The clinical utility of COL4A1 is highlighted by its positive association with decreased patient survival [137]. In vitro knockdown studies of COL4A2 in TNBC cell lines have been shown to decrease proliferation and increase apoptosis [138], emphasizing collagen type IV’s importance in maintaining the proliferative phenotype. Although generally less studied in breast cancer, collagen type X is understood to promote invasion in other cancer types through binding of DDR2 [142, 143] and has become increasingly acknowledged for its role in breast cancer progression. COL10A1 mRNA expression is reported to be elevated in DCIS and IBC compared to normal breast tissue. This coincides with increased COL10A1 expression found in breast cancer specimens (*n* = 125) compared to normal breast tissue (*n* = 18) from the Clinical Proteomic Tumor Analysis Consortium. Between DCIS and IBC cases, a comparison of COL10A1 expression in myofibroblasts and myoepithelial cells revealed increased expression in DCIS cases [144]. However, COL10A1 knockdown in tumor lines reduced invasion [139]. This suggests that cell-specific changes in COL10A1 expression can drive tumor progression and that increased expression in myoepithelial cells might have an anti-invasive effect. The prognostic value of the α_1_ chain of collagen type X has been underscored by its positive association with decreased overall and relapse-free survival. Furthermore, COL10A1 has been associated with increased CD4 + T-cells, CD8 + T-cells, and macrophages, which have aforementioned associations with high recurrence risk features in DCIS [145]. In addition to being positively correlated with other prognostic ECM proteins such as COL11A1 [140], serum levels of α_1_ chain of collagen types X and XI are elevated in both benign breast disease (*n* = 42) and breast cancers (*n* = 52) compared to healthy controls (*n* = 50) by ELISA assays [55, 140]. All in all, network-forming collagens have demonstrated an important role in mediating invasion; however, there is a paucity of studies to evaluate if these collagen types are linked to IBC progression in DCIS patients.

Fibrillar-associated collagens with interrupted triple helices (FACIT) have been increasingly investigated for their contribution to breast cancer transformation. FACIT collagens are defined by their triple helical domains separated by non-triple helical regions and are crucial in fibrillar collagen regulation [146]. Collagen type XII has been shown to be increasingly upregulated throughout the progressive stages of hyperplasia, adenoma, and metastatic adenocarcinoma in the spontaneous polyoma middle-T antigen (PyMT) genetically engineered mouse model (GEMM). Elevated COL12A1 expression was linked to increased tumor stiffness and changes in fiber structure, which were reversed with COL12A1 knockdown. In a TNBC patient cohort, increased immunohistochemical expression of the ⍺1 chain of collagen type XII was associated with decreased survival outcomes [147]. In addition to collagen type XII, a 22-patient study reported FACIT collagen, type XIX, and multiplexin collagen, type XV, to localize to the basement membrane in normal breast tissue. Within the study, the staining pattern was notably altered in invasive disease with the breakdown of the basement membrane as collagen types XV, XIX, IV, and laminin were within the stroma surrounding invading cells. Interestingly, half of the DCIS samples demonstrated loss of collagen types XIX and XV from the basement membrane [141]. This could reflect a difference in IBC progression risk; however, further investigation of FACIT collagens is necessary to understand their specific contribution to malignant transformation. In the context of invasion, FACIT collagens have been primarily studied in small human cohorts and murine models. To demonstrate clinical utility in DCIS risk stratification, the expression of FACIT collagens must be linked to invasive recurrence patterns in human cohorts.

Distinct from other collagens, collagen type VI (ColVI) forms a beaded microfilament suprastructure throughout the tissue microenvironment with major roles in cytoprotective function, control of differentiation, apoptosis, and autophagy [148]. ColVI is known to be secreted by adipocytes, an important constituent in the normal breast microenvironment [149]. In the context of breast cancer, the α3 chain of collagen type VI has been predominantly studied [150–152]. The α3 chain of collagen type VI has demonstrated positive immunohistochemical staining with progressive stages of the MMTV-PyMT transgenic mouse model [152]. Interestingly, the α3 chain of collagen type VI is proteolytically cleaved into the bioactive fragment, endotrophin, which has been implicated in breast cancer progression [151, 153]. PyMT murine models expressing endotrophin experienced higher tumor burdens and lung metastases compared to the controls. High endotrophin expressers led to increased levels of the angiogenic marker, HIF1α, the inflammatory marker, TNFα, and epithelial-mesenchymal transition markers with increased endothelial cell migration [150, 151]. There are currently few to no investigations on endotrophin in DCIS with the potential for invasive breast cancer progression.

Collagen type XVIII, a multiplexin type of collagen, also has a bioactive cleavage product, endostatin [115]. Endostatin has been demonstrated to have anti-tumorigenic effects which are thought to be mediated through its anti-angiogenic properties [154]. However, Guo et al. demonstrated that M1 macrophages partially contributed to its antitumorigenic effects. Using a co-culture system of RAW264.7 and 4T1 cells, overexpression of endostatin in RAW264.7 cells was discovered to reduce invasion and number of breast cancer cells. In vivo, endostatin was found to decrease tumor growth index over 15 days [155]. In a breast cancer study, Balasubramanian et al. demonstrated that high to moderate immunohistochemical staining for endostatin was associated with improved survival outcomes compared to patients with low expression [156]. Endostatin has been explored in phase I-III clinical trials often in combination with chemotherapies [157–160]. In the phase III clinical trial for use in breast cancer, a combination of docetaxel, epirubicin, and recombinant endostatin (*n* = 401) compared to docetaxel and epirubicin alone (*n* = 402) was found to achieve more complete, partial, and overall responses [158]. Although further mechanistic investigations are needed to understand its anti-tumorigenic effects, endostatin has therapeutic potential in invasive breast cancer with an unknown effect in the prevention of IBC recurrence in DCIS patients.

Many collagen types have demonstrated prognostic value in breast cancer and a role in breast cancer progression. However, the clinical integration of these collagen markers in DCIS risk stratification requires more studies in the context of the innate tissue microenvironment to validate their predictive value in IBC recurrence. The aforementioned studies highlight the predominance of transcriptional and global proteomic investigations into the collagen matrix in breast cancer. While this overarching view underscores the significance of collagen types, it does not elucidate how its discrete cellular domains and their post-translational regulation contribute to malignant transformation. Thus, a more detailed view of the site-specific post-translational modification of the collagen proteome is warranted in breast cancer.

**Table 1:** Collagen Types as Markers of Breast Cancer Progression

Collagen Type	Gene Name	Methods	Models/Database	Key Findings	Citation
MAJOR FIBRILLAR COLLAGENS					
**I**	**COL1A1**	Transcriptomics	TCGA	↑ in BC, worse survival in ER + BC	[124]
	**COL1A2**	Transcriptomics	TCGA	↑ in BC	[124]
**III**	**COL3A1**	IHC, knockdown	TNBC cell lines, TNBC human cohort	Invasion, proliferation	[130]
		IHC, IF, SHG, knockdown	TNBC and hormone-receptor cell lines, fibroblast cell lines, 3D culture system, human cohort, 4T1 murine model	Collagen alignment, ↓ in IBC compared to non-invasive BC, benign phenotype in breast epithelial cells, decreased metastasis, high COL3A1:COL1A1 associated with better survival outcomes	[129]
**MINOR FIBRILLAR COLLAGENS**					
**V**	**COL5A1**	Transcriptomics	TCGA	↑ in BC	[124]
		Knockdown, IHC, qPCR	TNBC cell lines, TNBC human cohort	Worse survival outcomes, chemoresistance, and M2 macrophage polarization	[121]
		IHC, qPCR	Fibroadenoma and IDC Human cohort	↑ in IDC compared to fibroadenoma	[122]
	**COL5A2**	Transcriptomics	TCGA	↑ in BC	[124]
**XI**	**COL11A1**	Transcriptomics	TCGA	↑ in BC	[124]
		IHC, ELISA	Human benign breast disease, IBC, and healthy controls	↑ in BC and in serum of BC patients	[55]
		IHC	Pure DCIS and mixed DCIS-IBC cohort	↑ in BC and invasive parts of mixed DCIS-IBC, total and invasive recurrence	[80]
		IHC	Human breast cancer and matched normal adjacent tissue	↓ in BC (specific isoforms)	[133]
		IHC	Breast cancer biopsies	↑ in IBC compared to DCIS	[134]
		IHC	Human breast invasive and in situ cancer cohort	↑ in IBC compared to in situ disease, microinvasion	[126]
**BEADED FILAMENT COLLAGENS**					
**VI**	**COL6A3**	IHC	GEMM	↑ with increasing stages of breast disease	[152]
		Knockout, overexpression, IHC, PCR	GEMM	Endotrophin: Metastasis, endothelial cell migration, proliferation, fibrosis	[151]
**NETWORK-FORMING COLLAGENS**					
**IV**	**-**	IHC	Mixed DCIS-IBC	Discontinuity in fraction of DCIS; Absent from basement membrane in IBC	[141]
	**COL4A1**	Knockdown, transcriptomics	HER2-positive cell line, Gene Expression Omnibus (GEO)	Proliferation, worse survival	[137]
	**COL4A2**	Knockdown	TNBC cell lines	Proliferation	[138]
**X**	**COL10A1**	IHC, ELISA	Human benign breast disease, IBC, and healthy controls	↑ in BC and in serum of BC patients	[55]
		Knockdown, invasion assays	TNBC and hormone receptor-positive cell lines	Invasion, proliferation	[139]
		Knockdown, invasion, transcriptomics, IHC	TNBC and hormone-receptor-positive cell lines, TRIMER database,	Invasion, increased immune infiltrate	[145]
**FIBRILLAR-ASSOCIATED COLLAGENS WITH INTERRUPTED TRIPLE HELICES (FACIT)**					
**XII**	**COL12A1**	LC-MS/MS identified, IHC, knockdown, overexpression	GEMM, TNBC human cohort	↑ in BC and benign breast disease, invasion, metastasis, & survival outcomes	[147]
		Transcriptomics	TCGA	↑ in BC	[124]
**XIX**	**COL19A1**	IHC	Mixed DCIS-IBC	[Altered localization dependent on pathology present	[141]
**MULTIPLEXIN COLLAGENS**					
**XV**	**COL15A1**	IHC	Mixed DCIS-IBC	Altered localization dependent on pathology present	[141]
**XVIII**	**COL18A1**	IHC	Breast cancer human cohort	Endostatin: better overall survival	[156]
		Overexpression	Murine breast cancer cell line	Endostatin: ↓ tumor growth and invasion	[155]

### Post-translational regulation of collagens in breast cancer

Hydroxylation [129] of proline residues is a key post-translational modification of collagen that modulates its structure and function (Table 2) [116, 117, 161–163]. Prolyl-4-hydroxylases form 4-hydroxyproline (Hyp), which contributes to collagen structural stability as well as in the opening and closing of its cell binding domains [116, 117]. This modification is most frequently observed at the second position in the triplet sequence (Pro-Hyp-Gly) and can increase binding affinity for cell receptors such as DDRs and integrins [164, 165]. Prolyl-4-hydroxylase can be constituted by either a β subunit dimer or as a tetramer consisting of two ⍺ and β subunits [166]. Notably, the ⍺ subunits have been predominantly studied in relation to cancer [167–170].

In breast cancer, decreased hydroxyproline content has been observed using colorimetric assays in the tumors compared to the normal adjacent tissue (*n* = 7) [171]. Despite the limited sample size and the non-specificity of the assay for collagens in this preliminary study, subsequent studies have shown altered expression of the enzymes responsible for these post-translational modifications, the prolyl hydroxylases, and have highlighted their role in breast cancer progression [172, 173].

Prolyl hydroxylase 4 subunit ⍺2, P4HA2, is linked to patient outcomes in IBC and DCIS. P4HA2 mRNA levels have been shown to be elevated in IBC compared to normal breast tissue and are associated with decreased overall survival probabilities. In the same study, P4HA2 expression was positively correlated with increased fibrillar collagen transcripts (COL1A1 and COL3A1) as well as network-forming collagen transcripts (COL4A1). This could be due to a positive feedback loop, in which hydroxylation of distinct collagen domains elicit β_1_ integrin-dependent signaling cascades that have been shown to facilitate further collagen deposition [100]. The in vitro investigation in the study demonstrated decreased invasion with P4HA2 knockdown and decreased proliferation with pharmacologic prolyl-4-hydroxylase inhibition. Congruently, in vivo orthotopic studies in SCID mice have reported reduced tumor growth, collagen deposition, and lung metastases in P4HA2 knockdown models compared to the controls [173]. In a study of pure DCIS (*n* = 776) and mixed DCIS-IBC (*n* = 239), high immunohistochemical expression of P4HA2 correlated with recurrence and high-risk histological features such as comedo necrosis, high nuclear grade, and HER2-positivity. Specifically, within the pure DCIS subset, high P4HA2 expression was linked to increased local ipsilateral recurrence [174]. Given hydroxylation of proline residues increases DDR and integrin binding affinity for collagen, elevated P4HA2 expression likely contributes to the activation of aforementioned EMT transcriptional programs that could support invasive recurrence [94–96].

Similar to P4HA2, prolyl-4-hydroxylase ⍺1 subunit (P4HA1) transcripts have also been found to be elevated in breast cancer compared to normal breast tissue and associated with decreased survival outcomes including recurrence-free survival [175]. Prolyl-4-hydroxylase subunit ⍺3 (P4HA3), was discovered to similarly be increased in breast cancer compared to normal breast tissue as was its fellow ⍺ subunits. Notably, higher P4HA3 expression was reported in the stroma than in tumor regions, which was not seen with P4HA1 and P4HA2 expression [141]. This could suggest stromal cell type-specific expression of P4HA3. Taken together, the ⍺ subunits of prolyl-4-hydroxylase are understood to be highly correlated with breast cancer demonstrating that post-translational alteration of the collagen matrix impacts the surrounding microenvironment to support tumor biology.

Although less studied, the multifunctional prolyl-4-hydroxylase subunit β, P4HB, has been reported to be elevated in breast cancer compared to normal breast tissue using TCGA transcriptomic data and in vitro systems (MCF-7, MDA-MD-231, SUM190PT, SK-BR-3). Interestingly, COL10A1 overexpression was found to increase P4HB expression. Silencing of P4H3 in COL10A1 overexpressing cell lines reduced invasion and migration rates similar to basal levels [139]. Thus, it seems that COL10A1 overexpressing cell lines might depend on P4H3 for appropriate post-translational regulation of collagen domains and to mediate invasion. While the β subunit has been less investigated in breast cancer, this study seems to support a similar upregulation as reported for the ⍺ subunits.

Despite the predominance of 4-hydroxyprolines within collagen, 3-hydroxyproline residues can occur [166]. The enzyme responsible for this modification, prolyl-3-hydroxylase, has not been as widely investigated as its prolyl-4-hydroxylase counterpart but has been associated with survival outcomes in breast cancer. A transcriptomic study of the TNBC GEO dataset (*n* = 118) revealed elevated P3H2 transcripts to be associated with more favorable survival outcomes [176]. A gene expression comparison between 13 breast cancer cell lines (MDA-MB231, MDA-MB361, MDA-MB436, MDA -MB468, MCF7, GI101, T47D, NCI, BT474, ZR75, SKBR3 and CAL51) demonstrated consistent P3H1 expression across cell lines. Interestingly, P3H2 and P3H3 had variable gene and protein expression among the cell lines tested. Certain cell lines exhibited no P3H2 and P3H3 expression, which was attributed to increased methylation. Expressing P3H2 and P3H3 in these non-expressing cell lines led to decreased proliferation [177]. Thus, this could suggest that P3H2 and P3H3 are selectively silenced to promote proliferation. Despite a small fraction of hydroxylated proline residues being 3-hydroxyprolines, these modifications seem to contribute to the regulation of the proliferative signaling pathways in certain breast cancers.

In summary, prolyl hydroxylases are crucial in modifying collagen and facilitate cell interactions that alter cell signaling. These studies demonstrate a role of prolyl hydroxylases in breast cancer and emphasize the importance of collagen post-translational regulation. While a global reduction of hydroxylation of proline residues has been reported to decrease invasion and metastasis in breast cancer, little is understood about how specific site modifications modulate cell signaling and contribute to the emergence and progression of breast cancer.

**Table 2:** Prolyl Hydroxylases Associated with Breast Cancer. Prolyl hydroxylase post-translationally modify collagen supporting its structure and function.

Enzyme Subunit	Methods	Models	Key Findings	Citation
**Prolyl-4-Hydroxylase Subunit** ⍺**1**	Transcriptomics, knockdown	Orthotopic murine models	↑ with poorer survival outcomes Proliferation, collagen deposition, and metastases	[175]
**Prolyl-4-Hydroxylase Subunit** ⍺**2**	Transcriptomics	TCGA	↑ in BC, with poorer outcomes, with recurrence-free survival	[173]
	IHC	DCIS and mixed DCIS-IBC human cohort	↑ with recurrence, comedo necrosis, high nuclear grade, and HER2-positivity	[174]
**Prolyl-4-Hydroxylase Subunit** ⍺**3**	Transcriptomics	TCGA	↑ in BC	[141]
**Prolyl-4-Hydroxylase Subunit β**	Transcriptomics, knockdown	TCGA Normal breast and BC cell lines	↑ in BC Invasion and migration	[139]
**Prolyl-3-Hydroxylase 2**	Overexpression	BC cell lines	↓ Proliferation	[177]
	Transcriptomics	TNBC GEO dataset	↑ with favorable survival	[176]
**Prolyl-3-Hydroxylase 3**	Overexpression	BC cell lines	↓ Proliferation	[177]

## Additional key extracellular matrix proteins and remodelers in DCIS

### Decorin associates with distinct breast pathologies

Decorin is an extracellular matrix proteoglycan known for its regulation of collagen fibrillogenesis [178, 179]. In vitro studies have demonstrated its anti-tumorigenic effects through transforming growth factor-β signaling [180] and decreased cell adhesion with recombinant decorin supplementation in specific breast cancer cell lines (BT474, T47D, SKBR3, HCT8/E11, SW480, and CaCo-2) [181]. Subsequent in vivo studies have reported decorin overexpression and recombinant decorin protein core administration to inhibit metastasis [182, 183]. Given its anti-metastatic and -tumorigenic effects, it is perhaps unsurprising that decorin has been found to have increased immunohistochemical expression in normal breast tissue (*n* = 97) compared to breast cancer (*n* = 187). Notably, decorin expression decreases progressively across the breast cancer stages with DCIS (*n* = 89) having increased decorin expression compared to IBC (*n* = 98) [184]. A subsequent study of 65 DCIS cases discovered patients with reduced periductal decorin expression to be at increased recurrence risk [185]. Its clinical relevance was further exhibited in a DCIS upstaging study (*n* = 153). Upstaging of DCIS can occur when only DCIS is detected in the biopsy, but IBC is also found at the time of surgical resection. Interestingly, a significantly larger portion of patients upstaged to IBC had decreased decorin expression [186]. Although decorin has differential expression across the breast cancer spectrum that appears to have clinical utility in DCIS risk stratification, it is not yet used clinically in DCIS.

### Tenascin C is linked to breast cancer invasion

Tenascin C is an extracellular glycoprotein that has been linked to many cancer types and their invasion [187–190]. In the context of breast cancer, it has been studied as an early indication of invasion. Initial studies reported that tenascin C expression in normal breast tissue increased over weeks 1 to 4 of the menstrual cycle but demonstrated consistent localization to the basement membrane and surrounding regions [191, 192]. Interestingly, DCIS demonstrated a similar expression pattern as normal breast tissue while IBC was found to have primarily stromal expression [191]. Increased staining intensities were noted in DCIS compared to normal breast tissue [192]. A subsequent study of 89 DCIS patients demonstrated a positive correlation between stromal or periductal tenascin C staining and microinvasion in addition to high-risk histological features such as comedo necrosis and high nuclear grade [193]. These findings coincided with the positive correlation between tenascin C staining and comedo necrosis found in the Iskaros et al. study. While Goepel et al. reported increased tenascin C expression in high-grade DCIS and IBC compared to benign breast diseases and normal breast tissue [194], the Iskaros et al. study did not support its positive correlation with nuclear grade [195]. Despite the lack of consensus over nuclear grade, increased stromal staining of tenascin C was consistently found to be positively correlated with invasive carcinoma [193, 195]. This suggests that tenascin C could be a crucial contributor to invasive transformation in breast cancer. Additional studies in invasive breast cancer have also linked its positivity at the invasive border to increased metastatic risk [196]. Given its association with invasion and high-risk histological features, tenascin C has shown prognostic value, which could be amenable to integration with other clinically utilized markers in the future following further validation in larger cohorts.

### Matrix metalloproteases correlate with breast cancer invasion

Matrix metalloproteases (MMPs) are endopeptidases that are well-characterized for their role in extracellular matrix remodeling. This family has a broad range of enzymatic activity against collagens, elastin, glycoproteins, and proteoglycans [197]. MMPs have been linked to invasion and metastases in many cancers [198–200]. In particular, the MMP class of gelatinases including MMP-2 and − 9 are considered to be facilitators of invasion through their degradation of basement membrane constituents [199, 201]. MMP-2 transcripts have been demonstrated to be increasingly upregulated from noninvasive to invasive breast cancer compared to benign breast diseases and normal breast [202]. In addition to MMP-2, Gonzalez et al. demonstrated increased MMP-7, -9, -13, and − 14 stromal fibroblast expression by immunohistochemistry near invasive regions compared to those surrounding DCIS lesions [203, 204]. Other studies have shown that significantly increased MMP-9 as well as MMP-26 protein expression in the DCIS lesion itself compared to atypical ductal hyperplasia, normal breast tissue, and IDC [205]. Thus, disparities in expression across studies could result from changes in localization that occur throughout breast cancer progression. Notably, expression of MMP-9, -11, and − 14 near IDC pathologies associated with shorter relapse-free survival. However, only MMP-11 expression in the DCIS component of mixed DCIS-IDC cases associated with shorter relapse-free survival [204]. This might suggest progressive changes in MMP expression with invasive transformation.

MMP-1, -8, and the aforementioned MMP-13 constitute the class of MMPs known as the collagenases due to their ability to cleave the triple helical structures of collagens type I, II, III, and V [206]. MMP-1 was found to have increased expression in DCIS compared the invasive component of mixed cases [203]. This might reflect differences in which DCIS and IBC cells remodel their local microenvironment and maintain their noninvasive and invasive phenotypes respectively. In DCIS, loss of MMP-8 has been reported specifically within the myoepithelial layer. In vitro studies of a DCIS-modified myoepithelial cell line (β6-1089) demonstrated that the restoration of active MMP-8 led to increased adhesion to and decreased migration in type I collagen, fibronectin, laminin I, tenascin C, and latency-associated peptide. Interestingly, overexpression of MMP-8 in a co-culture system with breast cancer cell lines (MDA-MB-231 and SUM159) decreased invasion [207]. MMP expression, therefore, changes from DCIS to IBC to remodel the extracellular matrix microenvironment in ways that promote invasion. Ideally, MMPs should be studied in conjunction with the ECM proteins they modify to better capture the pro-invasive microenvironment. It is perhaps this complexity that challenges the study of MMPs as biomarkers in DCIS (Table 3).

**Table 3:** Other ECM Proteins and Remodelers Linked to DCIS

ECM Protein	Methods	Models	Key Findings	Citation
**Decorin**	Overexpression	Chinese hamster ovary cells	Decorin binds TGF- β	[180]
	Recombinant protein supplementation	BT474, T47D, SKBR3, HCT8/E11, SW480, and CaCo-2	Reduced cell adhesion	[181]
	Overexpression	MDA-MB231	↓ tumorigenesis and bone metastasis	[182]
	Recombinant protein core supplementation	MTLn3	↓ lung metastasis	[183]
	IHC	Patient cohort	↓ in BC; ↓ in IBC compared to DCIS	[184]
	IHC	DCIS patient cohort	↓ in patients at increased risk for recurrence	[185]
	IHC	DCIS patient cohort	↓ in DCIS patients upstaged to IBC	[186]
**Tenascin C**	IHC	Normal breast, benign disease, DCIS, and IBC patient cohort	Localized to basement membrane area in normal breast and DCIS; ↑ in DCIS	[191]
	IHC	Normal breast, DCIS, and IBC patient cohort	Localized to basement membrane in normal breast	[192]
	IHC	DCIS patient cohort	↑ in IBC compared to DCIS, with microinvasion, comedo necrosis, and high nuclear grade	[193]
	IHC	Normal breast, benign disease, DCIS, and IBC patient cohort	↑ in high grade DCIS and IBC	[194]
	IHC	DCIS patient cohort	↑ in comedo necrosis DCIS, IBC	[195]
	IHC	IBC patient cohort	↑ in patients at increased risk for metastases	[196]
**Matrix metalloproteases**	In situ hybridization	Normal breast, benign disease, DCIS, and IBC patient cohort	↑ MMP-2 in DCIS and IBC	[202]
	IHC	DCIS, mixed DCIS-IDC, IDC cohort	↑ MMP-7 and − 9 in IDC compared to DCIS, ↑ MMP-1 in DCIS compared to invasive regions	[203]
	IHC	Normal breast, benign disease, DCIS, and IBC patient cohort	↑ MMP-2, -7, -9, -13, and − 14 near invasive regions; MMP-11 expression in the DCIS regions associated with shorter relapse-free survival	[204]
	IHC, in situ hybridization	DCIS, mixed DCIS-IDC cohort	↑ MMP-9 in DCIS compared to IDC	[205]
	IHC, adhesion and invasion assays	Normal breast and DCIS cohort, myoepithelial and breast cancer cell lines	↓ MMP-8 in DCIS compared to normal breast, ↓ MMP-8 led to increased invasion of breast cancer cell lines	[207]

## Perspective

The fact that DCIS has been studied for decades with still no apparent way to predict progression to disease highlights that different molecular viewpoints are required. Tissue mechanisms of the DCIS microenvironment are a composition of immune cells, fibroblasts, proliferative cells, and surrounding extracellular proteins that control gradients and fieldwide cell-cell communication. A cumulative point from the numerous studies detailed here is that the extracellular microenvironment has a significantly strong role in conversions of DCIS to IBC yet remains an understudied aspect of the disease. The extracellular microenvironment is unique as it is a protein composition with significant post-translational modifications. Details of critical protein domains, cell-interactive post-translational modifications, and field gradient composition thus cannot be completely reported by single cell transcriptomic studies nor by single or multiplexed antibody-driven studies. The major molecular knowledge gap into DCIS transitions is thus the lack of information on extracellular protein composition in combination with cellular status. Future directions require focusing on a holistic viewpoint that *combines* extracellular protein composition with single cell information that is ideally both transcriptomics and proteomics. Such holistic studies will be crucial in understanding key differences in lesion architypes, field connections between clonal populations and recurrence, integrity breaching of the myoepithelial membrane, and compositional secretion from progressively higher nuclear grade lesions. Currently, there is an uptake in digital pathology, pathology driven by artificial intelligence, with increasingly higher-speed instrumentation capable of attaining multiplexed expression patterns. With all of these advancements, it is not unrealistic to consider molecular pathology spanning both cellular and extracellular composition as a future clinical risk assessment for DCIS patients.

**Table:** 

Definitions List	
Ductal carcinoma in situ	A non-invasive breast pathology in which abnormal ductal cells are surrounded by an intact basement membrane. Although 70% of cases are classified as estrogen receptor-positive, DCIS can be classified by different hormone receptor expression [16]
Triple-negative breast cancer	A subtype of breast cancer characterized as lacking expression of estrogen receptor, progesterone receptor, and human epidermal growth factor receptor 2 expression.
Fiber alignment	The orientation of collagen fibers in relation to the tumor border.
Collagen regulation	The translational and post-translational regulation of collagen fibers.
Malignant transformation	The conversion from ductal carcinoma in situ to invasive breast cancer.

## Data Availability

The datasets used and/or analyzed during the current study are available from the corresponding author upon reasonable request.

## Abbreviations

AI: Artificial intelligence
CAFs: Cancer-associated fibroblasts
ColVI: Collagen type VI
DCIS: Ductal carcinoma in situ
ECM: Extracellular matrix
FACIT: Fibril-associated collagen with interrupted triple helices
GEMM: Genetically engineered mouse model
HYP: Hydroxylated proline
IBC: Invasive breast cancer
IDC: Invasive ductal carcinoma
MMP: Matrix metalloprotease
P4HA1: Prolyl-4-hydroxylase ⍺1 subunit
P4HA2: Prolyl-4-hydroxylase ⍺2 subunit
P4HA3: Prolyl-4-hydroxylase ⍺3 subunit
P4HB: Prolyl-4-hydroxylase β subunit
P3H1: Prolyl-3-hydroxylase subunit 1
P3H2: Prolyl-3-hydroxylase subunit 2
P3H3: Prolyl-3-hydroxylase subunit 3
PyMT: Spontaneous polyoma middle-T antigen
TACS: Tumor-associated collagen signature
TAMs: Tumor-associated macrophages
TILs: Tumor-infiltrating lymphocytes
TNBC: Triple-negative breast cancer
